# Triangular Assessment of the Etiology of Induced Abortion in Iran: A Qualitative Study

**DOI:** 10.5812/ircmj.9442

**Published:** 2013-11-05

**Authors:** Zahra Motaghi, Afsaneh Keramat, Mohammad Shariati, Masud Yunesian

**Affiliations:** 1 Department of Reproductive Health, Shahroud University of Medical Sciences, Shahroud, IR Iran; 2 Department of Community Medicine, Tehran University of Medical Sciences, Tehran, IR Iran; 3 Department of Environmental Health Engineering, Faculty of Public Health, Tehran University of Medical Sciences, Tehran, IR Iran

**Keywords:** Induced Abortion, Unwanted Pregnancy, Qualitative Research, Etiology

## Abstract

**Background:**

About 46 million induced abortions occur in the world annually. The studies have reported 80000 cases of induced abortions in Iran annually.

**Objectives:**

This qualitative study was conducted to identify the causes of unsafe abortion in Iran from the standpoint of three groups of experts, women with a history of abortion or unwanted pregnancy and service providers.

**Patients and Methods:**

A total of 72 in-depth semi structured interviews were conducted in 2012 in Tehran and Shahroud. After coordination with 8 experts, sampling from them was done using the Snowballing method in their offices. Sampling from 28 married and 10 engaged women with a history of unwanted pregnancy or unsafe abortion and 12 providers was done in health care centers and a in number of gynecologists’ and midwives’ offices. Sampling from women with a history of unwanted pregnancy or unsafe abortion such as single women, HIV positive women and drug users, and women who had sexual intercourse for money was started by referring to the social rehabilitation center for women and continued using the snowballing method due to difficulties in accessing them. Participants were from different ethnic groups including Fars, Gilaks, Mazandarani, Arab, Azerbaijani, and Lor. Content analysis was performed on collected data.

**Results:**

Based on the results of the interviews, participants have abortion for following reasons: 1. Wanted pregnancy (sub categories: fetal abnormalities, Concern about fetal health and lack of trust to prenatal diagnostic methods, Fetal sex, Lack of independent and free decision making regarding pregnancy in women, 2. Unwanted pregnancy (sub-categories: Socio-economic factors, Beliefs and feelings, Lack of information about family planning) 3. Predisposing factors (sub-categories: Lack of information on religious aspects of abortion, Easy access to easy abortion methods). Some people, despite having unwanted pregnancy due to social, economic, cultural and family grounds, continued their pregnancy and did not have an abortion for the following reasons: Religious beliefs, Beliefs (fear of punishment in the afterlife and believing in fate) , Attachment to the unborn baby, Influence of the other people’s opinions (physician, mother or spouse) Late diagnosis of pregnancy, Unsuccessful abortion attempts (Self-treatment, Unsuccessful medical abortion), Economic weakness and arbitrary treatment.

**Conclusions:**

In the present study, women who continued their pregnancy despite being unwanted were also interviewed. Although they had the same social, economic, cultural, and family problems as women with a history of unsafe abortion and had easy access to abortion, analysis showed that the difference in religious beliefs between the two groups was the most important factor that led women to choose two different approaches. The authors believe that in-depth analysis of people’s beliefs and opinions in this regard and correction of false beliefs plays a crucial role in decreasing the rate of unsafe abortion.

## 1. Background

About 46 million induced abortions occur in the world annually. About half of these abortions take place in developing countries which are mostly unsafe (1, 2). In 2010, Sousa et al. showed that nearly half of the abortion in Mexican women aged 15-55 years were induced and about a quarter of them were unsafe (3). There are no official statistics of induced abortion In Iran. However, various studies have reported 80 000 cases of induced abortions in Iran annually (4, 5). Studies have shown that serious complications such as maternal death, uterine rupture, and sepsis may occur after induced and unsafe abortion (6). In 2006, Grimes et al. reported that “an estimation shows that 68 000 women die from unsafe abortion every year, and also millions of women become injured which are mostly permanent” (7). Thus, unsafe abortion imposes heavy direct and indirect costs on families, as well as the health care system of each country. Each induced abortion costs about 53 to 143 USD in Mexico which may increase to more than 2343 USD in case of severe complications (8). The cost of an abortion is about 200 USD in Iran (9). Although unsafe abortion is very risky and costly, women have an abortion for many different reasons. Unintended pregnancy, economic class, low education, and living in poor neighborhoods are associated with abortion in Mexican women (3). Health Policy makers need the information of induced abortion to promote maternal health and reduce maternal mortality, which are the aims of MDGs (1). Therefore, this qualitative study was conducted to identify the causes of unsafe abortion in Iran from the standpoint of three groups of experts, women with a history of abortion or unwanted pregnancy and service providers. The aim of this study was to better understand the etiology of induced abortion by access to the inner world of the participants in order to suggest a hypothesis and to provide a questionnaire for a quantitative study in the second phase of this research. Induced abortion is a complex phenomenon. It is a dichotomy in decision making. A woman, in spite of having maternal feelings, chooses to abort her child. A qualitative study was a suitable method for discovering the reason and the quality of this phenomenon (10). Although some studies have been performed on the causes of induced abortions in Iran, this study was conducted in a triangulation fashion and the causes of unsafe abortions were examined from the standpoint of three groups of participants. The research question was asked in two senses including the second person (your reasons for abortion) and the third person (other people’s reasons for abortion) to collect more precise information. On the other hand, we interviewed people with similar conditions who continued their pregnancy without having an abortion and their views were explored.

In Iran, Induced abortion is allowed in cases such as saving the mother’s life or specific abnormalities in the fetus (5, 11, 12).

Moreover, health care centers provide services free of charge including family planning, and prenatal and postpartum care for target groups. Although these centers do not provide safe abortion services, some women who are not willing to continue their pregnancy and seek a reasonable solution for the its termination and need professional counseling in this regard refer to these centers while wealthy women refer to gynecologists.

Social rehabilitation centers for women provide free health, education, and welfare services to vulnerable women such as drug users, women who have sexual intercourse in exchange for money, the homeless women in Iran.

## 2. Objectives

In this study, we employed variation of sampling so that we interviewed participants with a history of unsafe abortion under various circumstances including married, single and engaged women, and sexual relations for money.

## 3. Patients and Methods

A qualitative method was used to obtain deep responses as to the cause of this phenomenon (10).

Induced abortion is a sensitive issue in Iran and therefore in-depth interviews were conducted to gain the trust of the participants. A total of 72 in-depth semi structured interviews were conducted until data was reached saturation of information, so “further interview provides no novel information, but redundancy of prior collected data. The interview included open and probe questions. The research question was asked in two senses including the second person (your reasons for abortion) and the third person (other people’s reasons for abortion) to collect more precise information.

Initial interviews served as guides for subsequent interviews. Thus, immediately after each interview, verbatim transcription was performed and analyses were conducted. If necessary, the interview questions were modified or new questions were added to the interview guide. The interview guide for the experts and service providers included a question about ways of accessing women with a history of unsafe abortion in addition to unsafe abortion causes which yielded useful results.

Variation of sampling including of Maximum variation sampling, Typical case sampling, Extreme (or deviant) case sampling, and Expert sampling were used in this study.

After coordination with experts, sampling from them was done using the Snowballing method in their offices.

Sampling from married and engaged women with a history of unwanted pregnancy or unsafe abortion and providers was done in health care centers and in a number of gynecologists’ and midwives’ offices in Tehran and shahroud.

Sampling from women with a history of unwanted pregnancy or unsafe abortion such as single women, HIV positive women, drug users, and women who had sexual intercourse for money was started by referring to the social rehabilitation center for women and continued using the snowballing method due to difficulties in accessing them.

Participants were from different ethnic groups including Fars, Gilaks, Mazandarani, Arab, Azerbaijani, and Lor. The interview was conducted when participants felt well and answered the questions with satisfaction.

Exclusion criteria were induced abortions done at a healthcare service by skillful provider such as physician for medical causes legally, gestational age after 22 weeks and the inability to participate in the interview such as psychiatric diseases.

Sampling was done in Tehran and Shahroud. Tehran is the largest city and the capital of Iran. About one seventh of the population of Iran lives in Tehran. Also, people from various nationalities and ethnic groups live in Tehran. Although Tehran reflects the culture of almost all ethnic groups and nationalities in Iran, the specific conditions of metropolitan cities may change people's views and culture. Shahroud is a small town located in Semnan Province in an area between the desert and the sea. There is not much immigration to this city and its inhabitants’ views and culture is not influenced by immigrants. Shahroud with a pristine culture represents most major Iranian cities, mainly cities located on the desert border.

### 3.1. Ethical Consideration

Ethical approval was obtained from the Medical Research Committee of Shahroud University of Medical Sciences. Consent was also obtained from the all associated administrations. Participants were included in the research voluntarily. Written consent was obtained from each participant to perform and record the interviews after being notified about the objective of the research and assuring them of the privacy and anonymity and confidentiality of the information and their right to refrain from the study in any phase.

Extreme attempts were performed to preserve privacy of the information during data collection by lack of access to the research team merely.

### 3.2. Participants’ Characteristics

1) Women with a history of unsafe abortion including of: 20 married women, 5 single women, 3 engaged women, 2 women who had sexual intercourse for money, 3 HIV positive and drug users.

2) Women with history of unwanted pregnancy: 3 single women, 1 women who had sexual intercourse for money, 7 engaged women, 8 married women.

3) 12 service providers

4) 8 experts 

### 3.3. Content Analysis

Content analysis was performed on collected data. Tapes of interviews were listened to several times and verbatim transcription was performed. Texts were carefully read to familiar with meanings and derived concepts. Initial codes were given to every meaning unit. Initial categories were derived from the combination of related initial codes. Central categories appeared from the integration of initial categories and summarization was done at every step to result fewer categories.

Data was collected and analyzed during the 12 months from May 2011 to May 2012.

## 4. Results

The data were collected from the 72 participants. Tables 1, 2, 3 summarize the Participants' characteristics. 

**Table 1.** Description of the Women with a History of induced Abortion and Women with History

Variable	Women With a History of Induced Abortion, No. (%)	Women With History of Unwanted Pregnancy, No. (%)
**Age, y**		
≤ 20	3 (9.09)	4 (21.5)
21 – 25	4 (12.12)	4 (21.5)
26 – 30	8 (24.24)	4 (21.5)
31 – 35	5 (15.15)	4 (21.5)
≥ 36	13 (39.39)	3 (15.8)
**Education**		
Illiterate	3 (9.09)	3 (15.8)
Grade school	13 (39.39)	2 (10.5)
High school	10 (30.3)	11 (57.9)
University	7 (21.2)	3 (15.8)
**Occupation**		
House wife	22 (66.7)	15 (45.4)
Student	2 (6.06)	-
Employee	2 (6.06)	3 (15.8)
Other	7 (21.2)	1 (5.3)
**Contraceptive method before induced abortion or unwanted pregnancy**		
IUD	1 (3.03)	1 (5.3)
Pill	1 (3.03)	-
Condom	5 (15.15)	2 (10.5)
Traditional	21 (63.6)	14
Unmet Need	5 (15.15)	2 (10.5)
**Marital status**		
Married	23 (69.7)	8 (42.1)
engaged	3 (9.09)	7 (21.2)
single	5 (15.15)	3 (15.8)
Others	2 (6.06)	1 (5.3)
**Gravid**		
1	3 (9.09)	6 (31.6)
2	6 (18.2)	9 (47.4)
3	7 (21.2)	0
≥ 4	17 (51.5)	4 (21.5)
**Parity**		
0	6 (18.2)	0
1	8 (24.2)	6 (31.6)
2	10 (30.3)	9 (47.4)
3	5 (15.15)	2 (10.5)
≥ 4	4 (12.12)	2 (10.5)
**Induced abortion**		
1	19 (57.6)	0
2	11 (33.3)	0
≥ 3	3 (9.09)	0

**Table 2.** Description of the Providers

Variable	No. (%)
**Age, y**	
20 - 30	3 (25)
31 - 40	4 (33.3)
≥ 41	5 (41.7)
**Education**	
≤ 16	8 (66.7)
≥ 22	4 (33.3)
**Occupation**	
Nurses and midwives	8 (66.7)
gynecologist	4 (33.3)

**Table 3.** Description of the Experts

Variable	No. (%)
**Education**	
18	3 (37.5)
≥ 22	5 (62.5)
**Proficiency**	
obstetrician	2 (25)
gynecologist	2 (25)
pediatrics	2 (25)
forensic medicine	1 (12.5)
lawyer	1 (12.5)
**Fields**	
policymaker	4 (50)
researcher	2 (25)
juridical	2 (25)

The results are divided in two major parts. The first part summarizes the causes of induced abortion from the standpoint of the three groups of participant including women with a history of induced abortion, service providers and experts. The second part shows the reasons why some women with unwanted pregnancy did not choose to have an abortion.

Part1: The causes of induced abortion

Based on the results of the interviews, participants have abortion for following reasons (Table 4). 

**Table 4.** The Causes of Induced Abortion based on the Results of the Interviews

Category	Subcategory	Inferior Category	Codes
**Wanted pregnancy**			
		Fetal abnormalities	Late diagnosis fetal abnormality (20%)
			Lack of awareness about abortion therapy lows (20%)
	fetal health		Minor fetal anomalies (20%)
		Concern about fetal health and lack of trust to prenatal diagnostic methods	use of drugs by wife or husband in the prenatal period (20%)
			History of fetal abnormally in the family (20%)
			Unintended pregnancy and no intake of folic acid (15%)
			Old pregnancy (15%)
			Maternal addiction (10%)
			Maternal disease (25%)
		Fetal sex	Tendency to girl (15%)
			Tendency to boy (15%)
	Cultural factors		Tendency to sort children sex (15%)
		Lack of independent and free decision making regarding pregnancy in women	Pressure from the spouse or partner made women choose abortion (39%)
			pressure from the family made women choose abortion (20%)
**Unwanted pregnancy**			
	Socio-economic factors	Economic factors	High costs of living (80%)
			Being a tenant (85%)
		The social, cultural and family status	Having small children (86%)
			Feeling embarrassed around their older children (70%)
			Having many children (87%)
			Having enough children (90%)
			non preparedness for having anothe baby (80%)
			Multiple pregnancy (8%)
			Concern about the future of the child (95%)
			Discord with spouse (65%)
			Addiction (80%)
			Pregnancy in unmarried women (75%)
			Pregnancy in temporary marriage (70%)
			Being divorced or widowed (50%)
			Pregnancy in the engagement period (90%)
			Career problems (40%)
			desire to continue education (%30)
			social class (%89)
			Welfare and tendency to progress (75%)
	Beliefs and feelings	Narcissism	When the question was asked in the third person "what is the cause of abortion by women?”
			Loss of freshness and comfort (50%)
			Welfare (50%)
			Loss of beauty and physical fitness (40%)
			Indolence and pleasure (20%)
		affect of others beliefs	Pregnancy during the engagement period (90%)
			Being blamed by others for having many children (85%)
		own beliefs	Impatience (65%)
			Having high expectations from life (56%)
			Believing that having children is futile (20%)
			Belonging to the higher social class with having less children (80%)
			Lack of side effects in medical methods of abortions (70%)
		Lack of information about family planning	Superficial information (70%)
			Lack of proper family planning counseling (70%)
			False beliefs (pregnancy during lactation and in the pre-menopause period is impossible) (80%)
**Predisposing factors **			
	The religious aspects of abortion	lack of information about the religious aspects of abortion	Those who were not aware of the religious aspects of abortion (60%)
			Those who were aware but did not care (80%)
			Those who were aware of the religious aspects of abortion and also cared but either had misunderstanding of the religious aspects of abortion in this regard or tried to bend the rules in their own interest. (70%)
	drug marketing	Easy access to easy abortion methods	Easy access to abortion methods (80%)
			Easy, available and safe medical methods of abortion (80%)

### 4.1. Wanted Pregnancy

A) Fetal abnormalities: responses of providers, experts and married women with a history of induced abortion showed that sometimes pregnancy is diagnosed late and as a result, fetal abnormalities are diagnosed when gestational age is more than four months. Since abortion is not authorized after four months gestational age, some mothers may choose to have an unsafe abortion themselves.

In some cases, it takes more than 4 months between diagnosing a fetal anomaly and obtaining legal abortion permission and sometimes mothers and their families are not even aware that legal therapeutic abortion is allowed and therefore may choose unsafe abortion. Sometimes, there are minor fetal anomalies and parents decide to have an unsafe abortion because abortion is not permitted in such anomalies.

Engaged and single women did mention these reasons. So fetal abnormality is not an important factor for abortion in these women and other reasons for induced abortion are more important to them.

B) Concern about fetal health and lack of trust to prenatal diagnostic methods: result of interviews showed that in cases such “use of drugs by wife or husband in the prenatal period, history of fetal abnormally in the family, unintended pregnancy and no intake of folic acid, old pregnancy, maternal addiction, and maternal disease” parents chose unsafe abortion because of concerns about fetal health. Engaged and single women did not mention these reasons. So these factors do not influence the decision for abortion in these groups.

A married woman (employee, education level: bachelor’s degree, her husband’s education level: master’s degree) stated that "my husband was using hormonal drugs for the treatment of hair fall when I became pregnant. I had an abortion because we were concerned about fetal health and my husband disagreed with amniocentesis.

C) Fetal sex: The results of the interview with service providers and experts showed some people had an abortion because of the fetal sex. Of course, engaged and single women did not mention this reason. Married women with a history of induced abortion also expressed this reason only when the question was asked in the third person as “why do women have an abortion?” but none of them mentioned they had an abortion for this reason. Of course, some of them stated that they regretted abortion when they found out that the fetal sex was their favorite.

D) Lack of independent and free decision making regarding pregnancy in women: Based on interview analysis, sometimes mothers wished to continue their pregnancy but pressure from the spouse, partner or the family made them choose abortion. This was especially true about the girls who were pregnant before marriage.

An unmarried woman with a history of abortion expressed “I had sex with my boyfriend. When I got pregnant; he became very angry and forced me to have an abortion”. 

Men sometimes forced their wives to have an abortion due to economic issues, social class, indolence and irresponsibility.

A married woman (employee, education level: bachelor’s degree) stated that” I loved to have children very much but my husband believed that only low class people had many children. I get pregnant for five times but my husband only agreed the first pregnancy. He made me abort other four pregnancies.”

### 4.2. Unwanted Pregnancy

All participants recognized unwanted pregnancy as the main reason of abortion. They stated that women with unwanted pregnancy had an abortion due to socioeconomic factors including “social, economic, cultural, and family factors” and beliefs and feelings including ” other people’s views, their own beliefs, and lack of correct information about family planning”.

A) Socio-economic factors: the results of the interviews indicated that socioeconomic factors influenced decision making regarding unsafe abortion. Economic problems were the reason for abortion in a large number of women. High costs of living for children, especially costs of education, were mentioned as an important factor for abortion. Because landlords do not rent out their houses to large families, being a tenant was one of the reasons for abortion, especially in big cities.

A married woman "I was very upset after I found out I was pregnant. Because I was a tenant and had a child and landlords do not rent out their houses to large families. I had to abort my child.

A married woman "My husband was addicted and unemployed. He said I had to abort because he did not want unnecessary guests."

Results indicated that the social, cultural and family status influenced decision regarding abortion. women had unsafe abortions for reasons including having small children, feeling embarrassed around their older children, having many children, having enough children, non preparedness for having another baby, multiple pregnancy, concern about the future of the child, discord with spouse, addiction, pregnancy in unmarried women, pregnancy in temporary marriage, being divorced or widowed, pregnancy in the engagement period, career problems, desire to continue education, social class, welfare and tendency to progress. Culture has a great impact on personal decisions in Iran. Decision about abortion in women who became pregnant during the engagement period depended on their culture.

Individuals in more traditional societies (from different ethnic groups including Fars, Gilaki, Mazandarani, Arab, Azerbaijani, and Lor) had an abortion to get rid of the blame because they were expected to become pregnant after marriage. It cost their lives in a few cases because they were not experienced like married people. They could not receive correct information about pregnancy and had an abortion due to fear of disclosure; therefore, they were often involved with non- professional swindlers. However, open-minded families (from different ethnic groups including Fars, Gilaks, Mazandarani, Arab, Azerbaijani, and Lor) performed the wedding ceremony sooner and let their daughters continue their pregnancy safely.

An expert “Last year, an engaged woman from a far city became pregnant. Despite she was educated and her husband wanted the child, due to their culture, she hid it from her family and had an abortion in unsafe and unhealthy conditions resulting in her death.

B) Beliefs and feelings: Analysis of interviews revealed that women’s beliefs and feelings affect their decision about abortion. “Loss of freshness and comfort, welfare, loss of beauty and physical fitness, indolence and pleasure” were reasons for abortion often expressed by providers and experts. Women with a history of abortion stated these reasons only when the question was asked in the third person "what is the cause of abortion by women", but none of them mentioned they had an abortion for any of those reasons. the results of the interviews with some participants in the three groups indicated beliefs such as “pregnancy during the engagement period, being blamed by others for having many children, impatience, having high expectations from life, believing that having children is futile, belonging to the higher social class with having less children, lack of side effects in medical methods of abortions” were reasons for abortion by women.

A married women “First, children are of no use. They do nothing to help their parents and only impose troubles and costs. They increase the population for no good reason. The fewer the children, the better the life.”

A married women "Nowadays, all the high class and educated people have only one child.”

C) Lack of information about family planning: Analysis of the interviews indicated that superficial information, lack of proper family planning counseling, and false beliefs (for example pregnancy during lactation and in the pre-menopause period is impossible) about family planning were reasons for unwanted pregnancy and abortion in women.

Many participants had little information about contraception before marriage. Pre-menopause women believed that they did not ovulate anymore and therefore did not use contraceptive methods. Lactating women or those who had administered DMPA did not use contraceptive methods since they believed they did not ovulate any longer. Others did not usually use contraception due to fear of complications and side effects of modern contraceptive methods. Some people imagined withdrawal to be the most reliable method and used abortion as a supplementary contraceptive method. Hence, most women with a history of abortion usually had an abortion more than once to terminate their pregnancy.

A married woman with three abortions (education level: high school diploma, employed) "My first child was born 15 years ago. I do not want more children and use the withdrawal method. I became pregnant three times in the past four years and aborted them. I try not to get pregnant again, but if I get pregnant, I will do the same. "

Some people did not use contraception because they received superficial and inaccurate counseling from their physicians or health care personnel.

### 4.3. Predisposing Factors

Lack of information about the religious aspects of abortion and easy access to easy methods of abortion are factors that facilitate abortion by women.

Lack of information on religious aspects of abortion: Analysis of interviews in the three groups of participants revealed that although abortion is forbidden in Islam, many people, even religious ones, misunderstood the religious aspects of abortion in Islam and performed it and therefore religion failed to prevent abortion in these individuals. Islam and religion were not concerning issues for unmarried and a lot of married women with a history of abortion.

Easy access to easy abortion methods: Analysis of interviews revealed that an important reason for abortion was that women with a history of induced abortion believed that medical methods of abortion were easy, available and relatively safe and reckoned that there was no reason for not using these methods. They even recommended abortion to their friends and abortion was something common to them and their friends.

Part 2: Some people, despite having unwanted pregnancy due to social, economic, cultural and family grounds, continued their pregnancy and did not have an abortion for the following reasons:

Religious beliefs: Married women who had deep religious beliefs refused to have an abortion and continued their pregnancy. However, none of the unmarried women mentioned religious beliefs as the reason for refusing abortion.

A married woman with unwanted pregnancy (post partum) “I had two children and I did not want another child. But because abortion is a sin and an act of murder, I did not have an abortion”

Beliefs: beliefs including fear of punishment in the afterlife and believing in fate prevented some women from having an abortion although their pregnancy was unwanted.

Attachment to the unborn baby: Someone women expressed that they did want the child at first but when they felt the fetus, they developed an emotional connection and attachment with it and decided to continue their pregnancy.

A married woman “I was very sad in start of my pregnancy and did not like it. When I went for ultrasound and felt its existence, I had a very pleasant feeling. I am now willing to tolerate any difficulty to give birth to a healthy child now”.

Influence of the other people’s opinions: Some married women refused to have an abortion and continued their pregnancy following their physician’s, mother’s or spouse’s advice.

A married woman “When I knew I was pregnant, I went to my doctor to have an abortion but the doctor advised me to keep it. He reminded me that I had only one child and told me that I would grow fond of the baby as time went by. Therefore I did not abort my child.”

Late diagnosis of pregnancy: this was often mentioned by pregnant women that were unmarried or engaged and continued their pregnancy. They found out they were pregnant late because they were inexperienced or had an underlying disease. Some women hid their pregnancy and families realized it very late.

A provider in a city stated: “Recently, a girl from one of the villages with severe abdominal pain came to me. Her Family though that she had appendicitis. When I examined her, she was in the second phase of delivery. She gave birth to a term baby.”

However, it was also noted by a few married women.

### 4.4. Unsuccessful Abortion Attempts

a) Self-treatment: some women said that their efforts to have an abortion were unsuccessful and therefore they continued their pregnancy.

b) Unsuccessful medical abortion: some women mentioned that despite receiving abortion services from professional health providers, their attempts were unsuccessful.

Economic weakness and arbitrary treatment: some women continued pregnancy due to lack of information about abortion and some because of inability to pay for abortion costs.

A married woman “When I found out I was pregnant, I took two shots of progesterone and four LD tablets to abort but they were useless. Then I went to some doctors for abortion but they charged at least 400-500 dollars and I did not have that money so I continued my pregnancy.”

## 5. Discussion

In this qualitative research, interviews were conducted about the causes of abortion with experts in the fields of health and law, service providers and women with a history of unsafe abortion.

In the present study, one of the reasons for abortion was maternal disease or concerns about fetal abnormalities. Studies in other countries also confirm these results (7, 13, 14).

The results of a similar study conducted by Visaria showed that induced abortion was done due to gender preferences. However, unlike his study in which boys were preferred (14), our participants preferred having equal number of boys and girls. Parents loved to have a boy as much as they loved to have a girl.

In the past, the son was preferred in Iran since he served as a labor force and the family authority symbol. Today, the role of members in a family has changed and therefore this belief has changed as well. Women often believe that daughters are kinder than sons and parents that have girls are considered to be prosperous. Usually, parents love their children, both boys and girls. In the study conducted by Visaria, mothers with female fetuses were forced to have an abortion through inflicting different types of physical or mental violence. In some cases, women who were accused of infidelity were violently forced to have an abortion (14).

Although in the present study, like some other studies in other countries, some women stated that they were forced to abort by their spouses or families (13), no woman reported forced abortion due to the female gender of the fetus. In the present study, unmarried women usually had an abortion due to the pressure from their partner or family.

In the present study, one of the most important reasons for abortion was economic issues, particularly being a tenant. Problems such as costs of living and education also had a significant influence on this decision. Economic problems have been shown to play an important role in making decision about abortion in other studies, as well (7, 13, 15).

Visaria (2004) also emphasized the role of economic problems in abortion in the Indian women. In his study, pregnancy and childbirth was as a threat to their career and fear of losing their job had an important role in having an abortion (14).

However, in the present study, abortion was not done due to fear of losing job probably because of better job security in Iran. In Mexico, induced abortion is more frequent in women of poor economic class,, low educated women, women with unintended pregnancy, women who have many children, and women living in deprived areas (3).

In our study, lack of attention to proper family planning counseling was an important factor in the occurrence of unwanted pregnancy and abortion. Just the same, limited access to family planning plays a major role in unwanted pregnancy and abortion in Mexico (3).

In Swedish adolescents, high-risk behaviors are important risk factors of unwanted pregnancy and abortion (16).

Inattention to correct use of contraceptive methods is an important factor in increasing the number of unwanted pregnancies and induced abortions in married and unmarried women in Pakistan (6).

Other reasons for abortion that were expressed in the present study were having many children, having small children, and feeling embarrassed around older children. In several studies in other countries, the design of the family planning programs, perfect age gap between children and no desire to have a large family have been stated as the causes of abortion (7, 13).

Another reason for abortion according to our study and the previous studies in other countries was the problems related to the wife’s or the husband’s employment and education (7, 13).

In the present study, some women believed that abortion was safe. They preferred the natural method of contraception and used abortion in case of pregnancy instead of modern contraceptive methods. Use of modern contraceptive methods is not common among Indian women. They have false beliefs about contraceptive methods and their complications. To them, abortion is clean, safe and accepted. They avoid modern contraceptive methods but are willing to have an abortion (14).

The results of the present study showed that some women had an abortion because of the problems related to their husbands such as his addiction, receiving no support from him, or discord with him. These reasons are not exclusive to Iranian women and also women in other countries terminate their pregnancy for these reasons (7, 13, 15).

In the present study, women who continued their pregnancy despite being unwanted were also interviewed. Although they had the same social, economic, cultural, and family problems as women with a history of unsafe abortion and had easy access to abortion, analysis showed that the difference in religious beliefs between the two groups was the most important factor that led women to choose two different approaches. People who had deep religious beliefs continued their pregnancy while women with a history of unsafe abortion belonged to one of the three groups including a) those who were not aware of the religious aspects of abortion, b) those who were aware but did not care and c) those who were aware of the religious aspects of abortion and also cared but either had misunderstanding in this regard or tried to bend the rules in their own interest.

In this study, women’s beliefs and feelings affected decision about abortion. It is noteworthy that in response to the research question “why did you have an abortion?”, in addition to socio-economic and cultural issues and maternal and fetal health, participants mentioned factors such as the stigma attached to pregnancy in the engagement period and feelings of inferiority due to others’ blames for having many children, pregnancy during the engagement period, being blamed by others for having many children, impatience, having high expectations from life, believing that having children is futile, belonging to the higher social class with having less children, lack of side effects in medical methods of abortions.

But when the research question was asked in third person "why do women have an abortion?”, in addition to the aforementioned responses, other factors such as loss of freshness and peace as a result of pregnancy and childbearing, damage to beauty and physical fitness, indolence and self-indulgence were expressed. It could be presumed that these reasons were important to them but were projected when asked in the third person.

In contrast, the few women who did not abort and continued their pregnancy mentioned fear of punishment in the afterlife as the main reason. The results showed no difference between participants' views in Tehran and Shahroud. The results of this qualitative study were used to provide a questionnaire and were carried out a quantitative study in the second phase of this research. This questionnaire can use to carry out some studies about induced abortion in Iran. The authors believe that in-depth analysis of people’s beliefs and opinions in this regard and correction of false beliefs plays a crucial role in decreasing the rate of unsafe abortion. The strong point of this study was variation of sampling to collect more precise information but purposive sampling was used in this qualitative study. So it isn't the representative ness of the sample.
